# Minimally invasive concretion-expulsion technique for primary canaliculitis: a prospective interventional study

**DOI:** 10.1186/s12886-025-04457-2

**Published:** 2025-11-05

**Authors:** Chengmei Wang, Changxu Liu, Lin Wang, Honglin Wang, Zuoxiang Pang

**Affiliations:** 1 Weifang Eye Hospital, Zhengda Guangming Eye Group, 139 Xingfujie, Weifang City, Shandong Province 261000 China; 2 Weifang Pepole’s Hospital, No. 151, Guangwen Street, Kuiwen District, Weifang City, Shandong Province China

**Keywords:** Primary canaliculitis, Minimally invasive canaliculotherapy, Lacrimal canaliculitis

## Abstract

**Purpose:**

To prospectively evaluate a minimally invasive concretion-expulsion technique for primary canaliculitis that combines systematic canalicular milking with intracanalicular antibiotic therapy, establishing its efficacy and safety as a viable non-surgical alternative.

**Materials and methods:**

This prospective interventional study was conducted over a 36-month period (June 2020–June 2023), enrolling patients with PCC treated using the concretion-expulsion technique and followed for at least 12 months. The procedure involved gentle massage along the conjunctival aspect of the canaliculus to express concretions and secretions from the common canaliculus to the canalicular ampulla, followed by mechanical evacuation through the punctum. This process was repeated, with adjunctive gatifloxacin gel instillation to dislodge residual debris, until no secretions were visible. Tobramycin-dexamethasone ointment was then applied. Demographic data, clinical features, and outcomes were analyzed statistically.

**Results:**

Ninety patients (90 eyes) were included, with a male-to-female ratio of 22.2% (20) to 77.8% (70) and a mean age of 66.27 ± 14.15 years. Laterality included 42 right (46.67%) and 48 left (53.33%) eyes, with involvement of the lower canaliculus in 57 cases (63.3%), upper canaliculus in 27 (30%), and both in 6 (6.7%). All patients exhibited increased ocular discharge, with common symptoms including redness(88.89%), epiphora (77.78%), swelling(55.55%), foreign body sensation (11.11%), and pain (6.67%). Concretions were present in 85 patients (94.44%), alongside signs such as mucopurulent discharge (44.44%), medial canthal swelling (38.89%), punctal eversion (“pouting”,33.33%), conjunctival hyperemia (27.78%), canalicular dilation (22.22%), and obstruction (11.11%). The mean symptom duration was 16 ± 11.06 months. A total of 66.67% were cured after one expulsion session, with a mean of 1.9 ± 0.83 sessions required overall. Subgroup analyses (dry eye status, age < 70 vs. ≥70 years, gender, symptom duration < 15 vs. ≥15 months) revealed no significant differences in treatment frequency (*P* > 0.05 for all). Complete resolution of symptoms and clinical signs was observed in all patients by the final follow-up.

**Conclusion:**

The concretion-expulsion technique is a minimally invasive, safe, and effective approach that preserves the anatomical and functional integrity of the punctum and canaliculus, representing a viable treatment option for primary canaliculitis.

**Supplementary Information:**

The online version contains supplementary material available at 10.1186/s12886-025-04457-2.

## Introduction

Canaliculitis, a chronic infection of the lacrimal canaliculi first described by von Graefe in 1854 [1], has an estimated annual incidence of 0.16 per 100,000 population [2]. It accounts for approximately 0.8% of lacrimal drainage infections and 2% of epiphora cases [3, 4]. Etiologically, it is classified into primary (91.59%) and secondary (8.41%) forms [5, 6]. Secondary canaliculitis is most commonly associated with the placement of punctal or canalicular plugs, whereas primary canaliculitis typically occurs in patients without prior canalicular surgery or trauma [7]. Although management of primary canaliculitis remains controversial, the removal of concretions and contents is critical for effective therapy [8].

Conservative strategies such as topical antibiotics or canalicular antibiotic irrigation have been reported [9–12]; however, these methods have high failure rates (approximately 65%–100%) and high recurrence rates (around 33%) [5, 13]. The primary reasons for failure include: The unique anatomy of the canaliculus, which is narrower and more tortuous compared to the nasolacrimal duct, creating a natural barrier to drug delivery; intraluminal concretions and secretions that shield bacteria and impede antibiotic access; Canaliculitis induced mucosal swelling, leading to canalicular obstruction; and Canalicular dacryoliths and inflammatory tissues further disrupt tear flow, perpetuating a cycle of stasis and infection [13, 14]. Ultimately, these factors contribute to resistance against both local and systemic antibiotics.

Complete removal of canalicular contents is therefore essential to prevent recurrence [8]. Historically, canaliculotomy with curettage served as the gold standard [13, 15]; however, traditional canaliculotomy (including punctum-involving incisions) is invasive and disrupts punctal and canalicular anatomy, leading to canalicular occlusion or lacrimal pump dysfunction in 20%–25% of patients, which can exacerbate epiphora [9, 15, 16]. Wang et al. [8] proposed a minimally invasive canaliculotomy, involving a 3-mm horizontal incision 3 mm medial to the punctum. A small curette (2 mm) was used to evacuate contents bidirectionally, followed by silicone tube intubation and suturing. This method achieved anatomical and functional success rates of 96.2% and 94.3%, respectively. In their control group, a 6–8 mm horizontal incision starting 2 mm medial to the punctum yielded anatomical and functional success rates of 92.7% and 81.8%, with the difference in functional success showing statistical significance. Preservation of canalicular morphology was associated with improved lacrimal pump function and reduced postoperative epiphora. Similarly, Liu et al. [17] retrospectively studied 35 patients (35 eyes) with primary canaliculitis treated using the same minimally invasive technique. Over a 1-year follow-up, anatomical and functional success rates were 97.1% and 91.4%, respectively. One patient developed canalicular obstruction 3 months postoperatively (1 month after tube removal), attributed to canalicular scarring. Despite these advancements, surgical interventions may still induce pump dysfunction or scarring, potentially worsening—rather than resolving—epiphora [5, 9, 12, 18].To address these limitations, here, we describe and prospectively validate a systematic, enhanced milking and expulsion technique focusing on mechanical concretions and secretions removal and targeted local therapy.

## Materials and methods

### Participants

This prospective interventional study enrolled 90 patients (90 eyes) with primary canaliculitis diagnosed at Weifang Eye Hospital between June 2020 and June 2023. All participants underwent concretion-expulsion therapy and completed at least 12 months of follow-up. The study was approved by the institutional ethics committee and conducted in accordance with the 2008 Declaration of Helsinki. Prior to inclusion, all subjects received detailed explanations of potential treatment-related risks and complications and provided written informed consent.

### Procedures

#### (1) Anesthesia preparation:

In the outpatient treatment room, 0.5% proparacaine hydrochloride eye drops were instilled into the conjunctival sac of the affected eye, followed by local infiltration anesthesia with lidocaine at the conjunctival fornix and caruncle.

#### (2) Massage-assisted concretion expulsion:

**Fig. 1:**
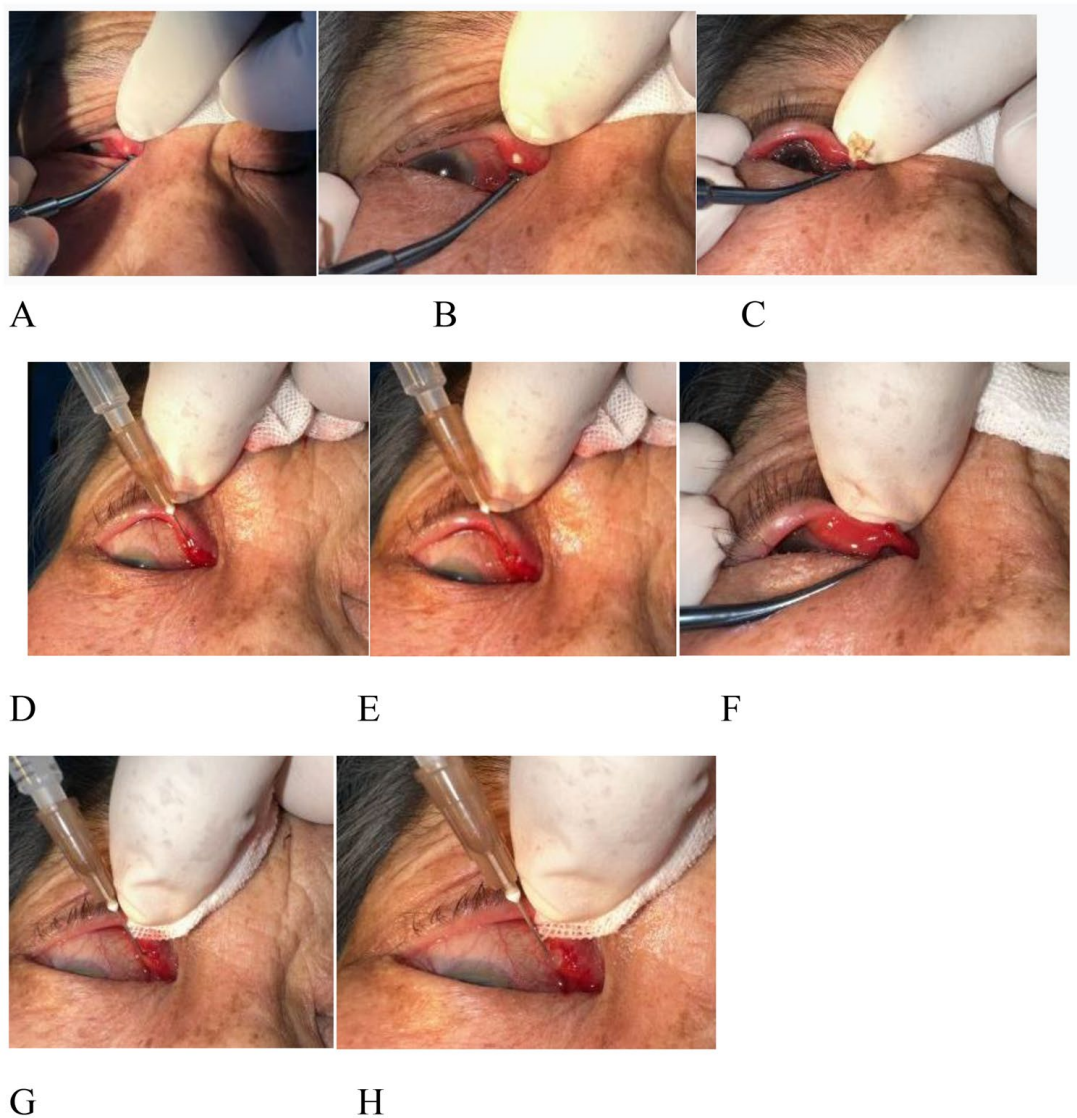
(**A**-**C**): Depiction of the process where a scleral depressor was used to perform massage and compression, moving from the common canaliculus towards the lacrimal punctum. (**D**-**F**): A 1-mL syringe fitted with a lacrimal irrigation cannula was utilized to instill gatifloxacin ophthalmic gel into the lacrimal canaliculus. During the gel instillation, overflow of the gel was observed from either the same or the contralateral lacrimal punctum. As shown in Image F, granulation tissue was seen extruding from the lacrimal punctum after the concretion-expulsion procedure. (**G**-**H**): Following the concretion-expulsion procedure, tobramycin-dexamethasone ophthalmic ointment was slowly injected into the lacrimal canaliculus while the needle was gradually withdrawn. Ointment overflow was noted from either the same or the opposite lacrimal punctum

The eyelid of the affected canaliculus was gently everted. The thumb was placed on the skin side of the diseased canaliculus, while the cylindrical end of a scleral depressor (glass rod) was gently slid along the conjunctival side of the canaliculus (Fig. 1A), applying linear massage from the medial canthus toward the temporal direction. This maneuver displaced concretions and secretions from the common canaliculus to the canalicular ampulla (Fig. 1B) and subsequently facilitated their expulsion through the punctum (Fig. 1C). Localized bulging of the canaliculus and punctual discharge of secretions were monitored as indicators of effective expulsion. Pressure was calibrated to avoid inadequate force (risking incomplete clearance) or excessive force (potentially traumatizing canalicular mucosa); patient feedback typically reported only mild discomfort.This maneuver was repeated until no further material was expressed, with immediate termination if significant pain occurred. (3) Gel-Assisted Debris Expulsion: A lacrimal irrigation cannula attached to a 1-mL syringe was used to inject approximately 0.2mL gatifloxacin ophthalmic gel into the canaliculus (Fig. 1D). Gel extrusion through the same or opposite punctum was observed during injection (Fig. 1E). Sequential massage and gel instillation were alternated until complete concretion clearance was achieved. In select cases, granulation tissue extrusion through the punctum was observed and recorded (Fig. 1F).

#### (3) Ointment injection:

Following debris removal, 0.1mL tobramycin-dexamethasone ophthalmic ointment was injected into the canaliculus via the same cannula (Fig. 1G). The cannula was withdrawn slowly during injection, allowing ointment extrusion through the same or adjacent punctum (Fig. 1H). Cannula positioning was adjusted according to individual canalicular anatomy to prevent mucosal injury, with strict avoidance of forceful insertion.

#### (4) Postoperative management:

Cold compresses were applied for 48 h, and patients received topical fourth-generation fluoroquinolones and low-concentration corticosteroids daily. Follow-up at 5–7 days evaluated healing parameters, with repeat sessions for residual symptoms.

### Outcome evaluation

**Canalicular dilation** was defined as visible swelling or bulging of the canalicular tract observed under slit-lamp examination.

**Canalicular obstruction** was confirmed by lacrimal irrigation, characterized by the complete reflux of fluid (frequently mixed with purulent material) from the punctum, indicating an inability to pass the fluid into the lacrimal sac.

#### Complete resolution

Disappearance of all clinical manifestations of canaliculitis (symptoms and slit-lamp findings) confirmed at final examination.

#### Recurrence

Reappearance of symptoms/signs ≥ 6 weeks after resolution.

Statistical analyses used χ² tests and two-proportion z-tests in SPSS 25.0, with *P* < 0.05 considered significant.

## Results

### Demographic and clinical characteristics

All patients completed a minimum 12-month follow-up.The cohort included 90 patients (90 eyes): 20 males (22.2%) and 70 females (77.8%), with a mean age of 66.27 ± 14.15 years (range: 18–98 years; median: 68 years). Laterality distribution was 42 right eyes (46.7%) and 48 left eyes (53.3%).Canalicular involvement included the upper canaliculus in 27 cases (30%), lower canaliculus in 57 (63.3%), and both in 6 (6.7%).Clinical presentations included increased ocular discharge in all patients, with conjunctival hyperemia observed in 80 cases (88.9%). Additional symptoms were epiphora (70 cases, 77.8%), medial canthal swelling (50 cases, 55.6%), foreign body sensation (10 cases, 11.1%), and pain (6 cases, 6.7%). Concretions were observed in 85 patients (94.44%), mucopurulent discharge in 40 (44.4%), medial canthal swelling in 35 (38.9%), punctal eversion (“pouting”) in 30 (33.3%), conjunctival hyperemia in 25 (27.8%), canalicular dilation in 20 (22.2%), and canalicular obstruction in 10 (11.1%). The mean symptom duration was 16 ± 11.06 months (range: 0.25–36 months), with details summarized in Table 1.

**Table 1:** Demographic characteristics in the present study

Parameters	Number and percentages *n* (%)
Sex	
Male	20 (22.2%)
Female	70 (77.8%)
Eye involve	
Right	42 (46.67%)
Light	48 (53.33%)
Age	66.27 ± 14.15 years (range: 18–98 years)
Canalicus involved	
Upper canaliculus	27 (30%)
Lower canaliculus	57 (63.3%)
Multiple canaliculus	6 (6.7%)
Presenting symptoms	
Discharge	90 (100%)
Redness	80 (88.89%)
Epiphora	70 (77.78%)
Swelling	50 (55.55%)
Foreign Body sensation	10 (11.11%)
Pain	6 (6.67%)
Presenting signs	
Mucopurulent discharge	40 (44.44%)
Medial canthal swelling	35 (38.89%)
Pouting punctum	30 (33.33%)
Conjunctival hyperemia	25 (27.78%)
Canalicular dilation	20 (22.22%)
Canalicular obstruction	10 (11.11%)
Presence of concretions	
Yes	85 (94.44%)
No	5 (5.56%)
Mean duration of complaints	16 ± 11.06 months (range: 0.25–36 months)
Number of concretion expulsions	
One	60 (66.67%)
Two	23 (25.56%)
Three	4 (4.44%)
Four	3 (3.33%)

### Treatment efficacy

**Fig. 2:**
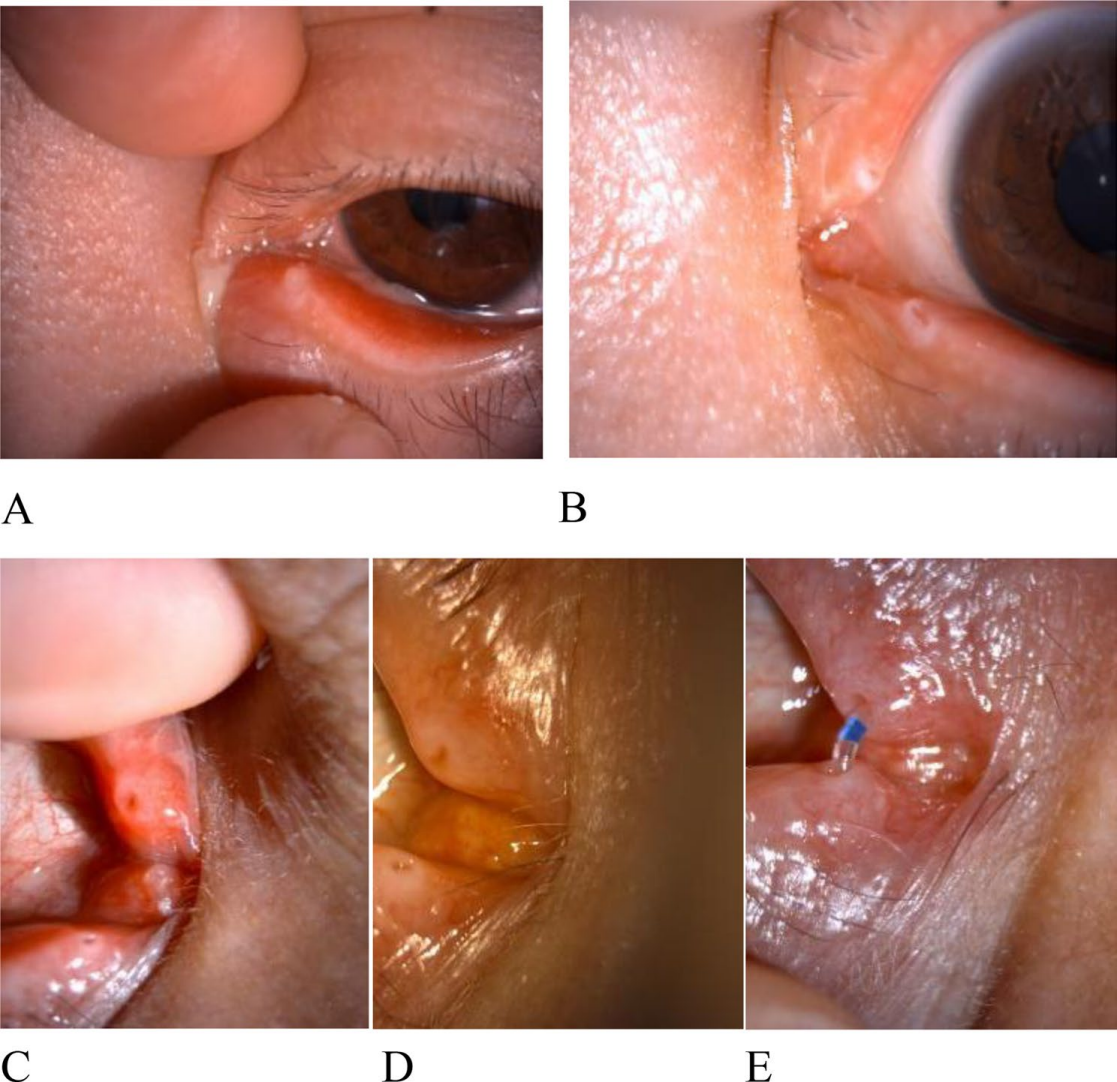
(**A**): Slit-lamp photograph of lower lacrimal canaliculitis, demonstrating canalicular erythema, punctal swelling with eversion, and purulent discharge at the punctum.(**B**): External ocular photograph of the same patient post-concretion-expulsion therapy, showing complete resolution of inflammatory signs.(**C**): Slit-lamp image of right upper canaliculitis, characterized by canalicular and punctal erythema with swelling.(**D**): Post-treatment slit-lamp photograph revealing full resolution of canaliculitis, with normalized punctal and canalicular appearance.(**E**): Post-treatment examination revealed lacrimal canalicular stenosis, with subsequent placement of a lacrimal stent

Sixty patients (66.7%) achieved cure after one session, 23 patients (25.56%) after two sessions, 4 (4.44%) after three sessions, and 3(3.33%) after four sessions, with a mean of 1.9 ± 0.83 sessions. Subgroup analyses comparing dry eye status, age (< 70 vs. ≥70 years), sex, and symptom duration (< 15 vs. ≥15 months) revealed no significant differences in treatment sessions required (Table 2). Clinical resolution of all symptoms and signs was achieved in 100% of patients. One patient (1.1%) experienced recurrence 6 months post-treatment, successfully managed with two additional expulsion sessions; no further recurrence occurred during the extended 12-month follow-up. Lacrimal patency was maintained in in 88 patients (97.8%); two patients (2.2%) developed distal canalicular stenosis, managed with temporary lacrimal stent placement (removed after 3 months) following symptom resolution, with subsequent imaging confirming restored lacrimal patency9(Fig. 2).

**Table 2:** Compares the number of concretions-expulsion

	Dry eye		Sex		Age		Disease duration	
Number	Dry eye	Non-dry eye	Male	Female	<70	≧70岁	<15月	≧15月
one	30	30	13	55	35	25	45	15
two	14	9	5	10	13	10	16	7
three	3	1	1	3	2	2	1	3
four	3	0	1	2	0	3	2	1
p value	c2=0.426，df=1，P=0.259		c2=1.67，df=3，P≈ 0.643		c2=3.99，df=3，P=0.262		c2=4.69，df=3，P=0.196	

## Discussion

This study cohort primarily consisted of middle-aged and elderly patients, a demographic tendency potentially explained by age-related shallowing of the lacrimal punctum that impairs drainage and increases infection risk [19]. Female patients accounted for 77.8% of the cohort, consistent with previous reports. This gender disparity may stem from menopausal hormonal changes in middle-aged and elderly women, which reduce tear production and weaken resistance to infections [9, 20]. Prolonged cosmetic use and household exposures (e.g.cooking fumes) may further contribute to canalicular obstruction and bacterial growth. The study also revealed a higher proportion of unilateral and lower canaliculitis cases. Research suggests that the susceptibility of the lower lacrimal canaliculus to infection is linked to gravity and its anatomical structure [21]. The lower canaliculus adopts an almost horizontal orientation and is longer than the upper canaliculus, allowing gravity to facilitate bacterial accumulation. Patients in this study experienced prolonged delays between symptom onset and definitive diagnosis of canaliculitis. Since its initial description by von Graefe in 1854, lacrimal canaliculitis remains frequently misdiagnosed or delayed (45%–100%) [22]. Primary canaliculitis is often mistaken for chronic conjunctivitis, chronic dacryocystitis, eczema, or infected chalazion [23]. Kaliki et al. [9] reported a median diagnostic delay of 6 months (range: 1–60) for primary canaliculitis. The condition severely impacts patients’ quality of life, with persistent epiphora and purulent discharge limiting daily social and occupational activities. To address diagnostic challenges, Singh M et al. [24] proposed a diagnostic “clinical tetrad”: medial eyelid edema, pouting and hyperemia of the lacrimal punctum, yellowish canalicular hue, canalicular distention with expressible discharge. Some scholars consider punctal ectropion or protrusion as characteristic signs of canaliculitis [25]. While ultrasound biomicroscopy (UBM) and lacrimal endoscopy have been suggested for diagnosis [18, 26], these require specialized equipment and technical expertise. In most cases, a detailed clinical examination suffices for diagnosis [9, 11].

The literature contains limited detailed descriptions of laboratory examinations for canaliculitis. Microbiological profiles in canaliculitis vary widely, with culture positivity rates ranging from 11% to 91% [9, 15, 16, 18, 27, 28]. Actinomyces was historically recognized as the most common pathogen in primary canaliculitis. While Actinomyces was historically considered predominant, recent evidence indicates Streptococcus and Staphylococcus are more common, often in polymicrobial infections [6, 29]. Actinomyces are filamentous Gram-positive anaerobic bacilli with fungal hyphae-like morphology, existing singly, in pairs, or chain formations [30]. Over 30 Actinomyces species have been identified to date. These organisms normally reside as commensal flora in human oropharynx, gastrointestinal tract, and female genital tract. Risk factors for Actinomyces infections include poor oral hygiene, oral mucosal trauma, diabetes mellitus, immunosuppression, alcoholism, and malnutrition [31, 32]. Chronic suppurative and granulomatous infections may occur at various anatomical sites when mucosal barriers are compromised [33]. Although ocular Actinomycosis remains relatively rare, Actinomyces species have been isolated from cases of endophthalmitis, keratitis, orbital cellulitis, dacryoadenitis, dacryocystitis, and canaliculitis [34–38]. Dry eye syndrome is a significant predisposing factor, while lacrimal duct stenosis and anatomical anomalies may promote tear stagnation, facilitating bacterial growth and concretion formation [24, 39, 40]. Histopathological examinations by Repp et al. support the strong association between Actinomyces, canaliculitis, and canaliculith formation [41]. Characteristic sulfur granules, though pathognomonic for Actinomycosis, are not exclusive to this condition and represent microbial aggregates mixed with inflammatory cell debris [42, 43].Discrepancies in microbial profiles among canaliculitis patients across studies may relate to specimen contamination and varying culture conditions [7]. Nonetheless, consistent patterns emerge, with Staphylococcus, Streptococcus, and Actinomyces representing the most frequently isolated pathogens [10, 29]. In a study of 52 cases by Zhang et al. [27], 60 bacterial isolates demonstrated highest susceptibility to fluoroquinolones, particularly gatifloxacin, supporting its use as first-line empiric therapy. Accordingly, all patients in our study received topical fourth-generation fluoroquinolone eye drops following concretion expulsion.

In 2015, Xu Jianjiang et al. [10] introduced intracanalicular antibiotic ointment infiltration (IOI) as a minimally invasive treatment for canaliculitis, demonstrating that sustained ointment retention enhances local drug concentration and bioavailability, potentially avoiding surgery. However, their technique required specialized catheters and an operating room. In 2020, Alam et al. [44] later modified the protocol by tailoring antibiotic selection to susceptibility testing, using standard lacrimal catheters with repeated administrations based on severity. Their cohort of 24 patients showed symptom resolution in all cases, with one recurrence at 12 months; lacrimal patency was maintained in 87.5%, though 4.2% developed canalicular obstructions.Our approach aligns with prior studies involving intracanalicular ointment instillation but emphasizes systematic removal of concretions and secretions as the core therapeutic strategy. The procedure begins with mechanical expression of canalicular contents using a scleral depressor.This is followed by instillation of 0.2 mL gatifloxacin ophthalmic gel, which not only flushes adherent concretions from the canalicular mucosa but also lubricates the lumen to facilitate subsequent expulsion maneuvers. In select cases, repeated expulsions resulted in extrusion of granulation tissue via the punctum. After thorough evacuation, tobramycin-dexamethasone ointment is instilled to provide prolonged anti-infective and anti-inflammatory effects. Punctal stenosis, when present, was gently dilated to ensure access while avoiding iatrogenic laceration.

The procedural goal of complete concretion removal is critical: 66.7% of patients achieved full resolution after a single session, with most remaining cases resolving after two or three treatments. We consider the efficient concretion-expulsion technique to be a key factor contributing to the higher cure rate in this study. Furthermore, the removal of concretions and secretions facilitates tear drainage, and the continuous tear flow maintains an oxygen-rich environment. The oxygen dissolved in tears helps eliminate infections while enabling local antibiotics to reach the infected sites and maintain adequate concentrations [18, 45]. In cases with residual canalicular stenosis after infection resolution, temporary stent implantation provides dual benefits: improving tear drainage and preventing adhesive obstruction through mechanical lumenal support [46].

We contextualized our technique against other minimally invasive options reported in the literature. For instance, the punctum-sparing mini-invasive canaliculotomy described by Wang et al. [8] and Liu et al. [17], while achieving high success rates, still requires a skin or conjunctival incision, carrying inherent risks of damaging the orbicularis muscle and postoperative scarring. In contrast, the intracanalicular ointment infiltration (IOI) technique reported by Xu et al. [10] and Alam et al. [44] is incision-free; however, their described techniques relied on specialized catheters or operating room settings, and focused primarily on drug delivery without emphasizing systematic mechanical removal of concretions. The advantage of our technique lies in its incision-free nature, feasibility in an outpatient setting, and the active ‘massage-gel irrigation’ step aimed at thoroughly evacuating the concretions that are central to the persistent infection, potentially contributing to the higher single-session success rate and lower recurrence observed. Certainly, its limitations include dependence on operator skill and potentially reduced efficacy in advanced cases with severe canalicular obstruction.

Subgroup comparisons were performed to evaluate the number of required concretion-expulsion sessions across dry eye status, sex, age (< 70 vs. ≥70 years), and symptom duration (< 15 vs. ≥15 months). Statistical analysis revealed no significant differences in treatment frequency among groups (*P* > 0.05 for all comparisons). A non-significant trend toward increased session requirements was observed in patients aged ≥ 70 years and those with dry eye disease compared to their counterparts. However, this finding should be interpreted with caution due to limited sample sizes in individual subgroups, which may have reduced the power to detect true associations.

In the study by Xu Jianjiang et al. [7], one patient experienced lacrimal canaliculus tear during ointment injection, but no lacrimal canaliculus obstruction was observed during the 2-month follow-up. In contrast, Alam et al. [44] reported three patients with lacrimal canaliculus obstruction, though it remained uncertain whether this resulted from the primary disease itself or wall damage caused during canalicular drug injection. However, studies have shown that residual ointment (whose main carriers include wax and mineral oil) may induce foreign body reactions, lipogranuloma, or paraffinoma formation [47]. In this study, all procedures were performed by experienced specialists, and no intraoperative complications such as canalicular tear occurred. Throughout the 12-month follow-up, we specifically monitored for delayed adverse effects potentially related to the ointment, such as lipogranuloma or foreign body reactions [47]. Reassuringly, no such signs were observed in any patient. We attribute this to the controlled technique of slowly injecting the ointment and allowing its expressible excess to exit via the punctum, minimizing significant residual retention within the lumen. Nevertheless, awareness of this potential risk remains crucial in clinical practice.

Although the technique achieved resolution in all patients in our cohort, it is important to acknowledge its potential limitations in managing refractory canaliculitis associated with complex anatomical factors, such as a lacrimal canalicular diverticulum. While such cases were not encountered in the present series, traditional surgical approaches like canaliculotomy may still be required in clinical practice if minimally invasive expression proves ineffective after multiple attempts. Although such data are valuable for understanding the pathogenic profile, this was not pursued routinely due to clinical practicalities and the study’s primary aims. Future studies incorporating systematic microbiological evaluation are warranted. Additional limitations of this study include the lack of a control group, potential selection bias, and a relatively short follow-up period. However, this minimally invasive technique demonstrates significant feasibility for implementation in primary care hospitals or among different operators.

## Conclusion

The concretion-expulsion technique represents an effective, minimally invasive, and safe treatment option for primary lacrimal canaliculitis that obviates the need for traditional surgical intervention in many cases. This study integrated manual massage, antibiotic gel irrigation, and ointment instillation into a standardized, reproducible sequential protocol, and being the frirst to validate its high efficacy and safty through a large-scale prospective study. By minimizing damage to the canalicular structure and function, it offers a viable alternative to conservative medical therapy and surgical intervention, potentially filling a gap in the current treatment paradigm. However, further prospective, randomized, controlled trials with extended follow-up are necessary to define the optimal patient population and indications for this technique.

## Supplementary Information

Below is the link to the electronic supplementary material.

Supplementary Material 1

## Data Availability

The datasets used and/or analysed during the current study are available from the corresponding author on reasonable request.
